# The Facial Appearance of CEOs: Faces Signal Selection but Not Performance

**DOI:** 10.1371/journal.pone.0159950

**Published:** 2016-07-27

**Authors:** Janka I. Stoker, Harry Garretsen, Luuk J. Spreeuwers

**Affiliations:** 1 Faculty of Economics and Business, University of Groningen, Groningen, The Netherlands; 2 SCS—Services Cyber Security and Safety Department of Electrical Engineering, University of Twente, Enschede, The Netherlands; Goethe-Universitat Frankfurt am Main, GERMANY

## Abstract

Research overwhelmingly shows that facial appearance predicts leader selection. However, the evidence on the relevance of faces for actual leader ability and consequently performance is inconclusive. By using a state-of-the-art, objective measure for face recognition, we test the predictive value of CEOs’ faces for firm performance in a large sample of faces. We first compare the faces of Fortune500 CEOs with those of US citizens and professors. We find clear confirmation that CEOs do look different when compared to citizens or professors, replicating the finding that faces matter for selection. More importantly, we also find that faces of CEOs of top performing firms do not differ from other CEOs. Based on our advanced face recognition method, our results suggest that facial appearance matters for leader selection but that it does not do so for leader performance.

## Introduction

The relevance of facial appearance for the selection of leaders, albeit CEOs or politicians, is by now well established [1]. Individuals whose faces signal competence and dominance are significantly more often chosen for a leadership position than individuals who look less competent and dominant [2–3]. However, whether facial appearance also signals leader ability and consequently actual performance is less clear. Results are especially contradictory on the relationship between CEOs’ faces and firm performance. On the one hand, ratings of CEO leadership qualities are found to positively relate to firm profits [4–6]. On the other hand, other authors found no significant relationship between facial traits of CEOs and performance [7].

More fundamentally, and based on a survey of all the evidence, it can be concluded [3] that social attributions from faces are much less accurate than previously thought. Accordingly, several scholars [8] even warn for the danger of ‘face-ism’, that is the overreliance on the accuracy of face-based inferences. The origin of ‘face-ism’ mainly lies in the way facial features are typically measured. Commonly, respondents are asked to give their personal assessment of pictures of alleged leaders. Most often in a lab setting, this may or may not concern actual politicians or CEOs [9–11]. Upon seeing the various leader’ faces, respondents either choose the leader [12], or score these faces on a number of variables that pertain to the leader’s abilities, like dominance, likeability, and trustworthiness [4].

We argue that research on the role of facial appearance could strongly benefit from an objective, and more advanced facial measure. We agree with Todorov and his colleagues [13] that especially for the relationship between leadership and performance we need to use more data-driven approaches to investigate the role of facial features. Against this background, and in order to advance the knowledge on the relationship between CEOs’ faces and firm performance, we use an objective instrument that is not suspect to the inaccuracy of human judgement. Up till now, the facial width-to-height ratio (fWHR) has been used as the main objective measure [6]. More recently, mouth width has been added as an objective facial feature [14]. However, these predetermined and one-dimensional measures have several shortcomings, since they are superimposed upon the data, and allow faces to matter only on this one given measure, like the fWHR. Moreover, there have been failures to replicate findings from these one-dimensional measures with respect to simple objective outcome variables [3], although a recent meta-analysis shows some evidence for the relationship between the fWHR and working context behaviors [15]. Therefore, in the biometric application of facial recognition, purely geometrically based methods like fWHR were applied early on only [16], but in this field have nowadays been replaced by approaches that take the appearance of the whole face into account [17] through statistical modelling of the facial appearance [18]. Based on machine learning principles, these modern facial recognition methods are instruments where the way possible patterns would matter is not given by one measure, but is based upon the data itself.

The main aim of our paper is to test the predictive value of CEOs’ faces for firm performance, by using a state-of-the-art objective measure for face recognition in a large sample of Fortune500 firms. We will compare CEOs’ faces of relatively good performing firms with faces from CEOs who are leading firms that perform less well, with the Likelihood Ratio, based on a statistical analysis using Principal Component Analysis (PCA) and Linear Discriminant Analysis (LDA) [19–20]. Unlike ‘traditional’ logistic regressions or ANOVA’s, LDA allows the separation of groups based on linear combinations of variables. This is the main reason why LDA is a standard approach in statistical classification literature. Importantly, next to the application of this advanced measure, our study differs in two ways from previous research.

First, whereas earlier studies focus only on *within*-group comparison of CEOs to assess the relevance of facial appearance (for a notable exception see [21]), our paper starts by analysing the general distinctiveness of CEOs faces. We compare CEOs with a sample of citizens to test whether faces of CEOs are indeed different. Moreover, we compare CEOs’ faces with those of university professors, since a possible significant difference between CEOs and citizens might be related to education or socio-economic status.

Second, it is the first time that such large samples are used, both in terms of the CEO sample itself, and the combined sample of citizens and professors. This enables better and more varied between ánd within group comparisons, which is a precondition for any robust study of both the selection and performance effect of faces. A significant difference between groups of CEOs and non-CEOs would clearly suggest a selection effect. Hereafter, by looking *within* the group of CEOs to investigate whether it is possible to differentiate between CEOs’ faces of good and bad performing firms, we can determine whether facial characteristics of a CEO relate to actual firm performance.

### Facial appearance, selection and performance

In selection processes, there is abundant evidence that stereotyping plays a strong role. Characteristics like gender [22], race [23] and physical attractiveness [24] impact perceptions of job suitability such that hiring, promotion and also reward decisions are strongly determined by both negative and positive stereotypical beliefs [7]. More specifically, these processes of stereotyping also include occupational stereotyping: there are facial stereotypes of various occupations (such as nurses or bankers), and people who look like this facial stereotype have a higher change of getting selected [25–26]. So first impressions, and in particular facial characteristics, influence these selection decisions [27].

These processes have also been shown to apply to leadership positions. The social information processing (SIP) theory [28] explains how information overload and task demands activate automatic information processing based on leadership schemata. Having a face that is mature (that is, not baby-faced) and attractive [29] is consistent with the expectations people have from a prototypical leader, which implies that people with mature and attractive faces appear to possess characteristics that would be beneficial for leading a business. Consequently, this perception of a prototypical leader triggers the attribution of leader-like traits and behaviors, leading to favorable selection outcomes and subsequently the acquisition of leadership positions [30–31]. And even *within* the domain of leadership positions, humans can accurately identify military, business and sports leaders from their faces [32].

But, although people can assess if a picture showing a face is indeed the face of a leader, and even a certain type of leader, it does not automatically imply that people in general are able to accurately deduct actual leadership qualities and consequently performance from facial appearance [32]. There are both strong advocates and opponents for the existence of such a relationship between facial appearance and actual performance. Supporters can be found in the field of evolutionary psychologists. Taking an evolutionary approach, these researchers suggest that physical characteristics as for instance measured by the fWHR, relate to leaders’ effectiveness [33–34]. The fWHR is a sexually dimorphic trait and—among men—a greater fWHR is found to be associated with more aggressive behavior [35] or more unethical behavior [36]. Note however that the status of fWHR as a sexually dimorphic trait is not undisputed [37]. One of the explanations for the suggested relationship between fWHR and performance is that a greater fWHR relates to showing aggressive behaviors, which in turn is associated with feelings of power [36], and these feelings of power subsequently have all kinds of positive outcomes, such as action taking, optimism and abstract thinking [38]. Based on this line of reasoning, in a study among 55 Fortune500 firms it was shown that firms with a CEOs with a larger fWHR have a superior financial performance [6]. In a similar vein, Rule and Ambady show that there is a relationship between facial characteristics, personality traits of both male and female CEOs and firm profits [4, 9], since traits like competence, trustworthiness [39] and dominance [40] can be reliably derived from facial appearance.

However, scholars who doubt the existence of a relationship between leader’ faces and performance argue that the relationship between faces and selection merely reflects biased human perception and is not an accurate inference of real or actual leadership qualities [8, 32, 41]. This position is supported by an empirical study showing that the causality of the relationship is reversed. In experiments with nearly 2000 subjects it was found that CEOs who looked more competent were hired by companies that already performed well [7].

While acknowledging that first impressions of faces are important in all kind of social interactions including the selection of leaders, the contradictory findings up till now suggest that one should be a lot more careful in concluding that these features are also strong predictors of actual firm performance and hence of the effectiveness of leaders. Clearly more research is needed. More importantly, we argue that the existing literature on facial inferences as predictors of leader’s success is limited due to the actual measurement of these facial features; following the call for the use of more data-driven approaches [13], we investigate the relationship between CEOs faces and firm performance by using an objective instrument that is not suspect to the inaccuracy of human judgement.

## Method

In biometric face comparison, facial images are compared in order to find out if they are recordings of the same person. In this paper, we want to investigate if a facial image belongs to one of two *classes* of persons, namely the class of US CEOs and a benchmark group. Next to a benchmark group of an ‘average’ US citizen, we also obtained photos to construct a class of US professors. We decided to add this ‘extra’ benchmark group, because the occupation of professor shares certain characteristics with the position of a CEO, like educational background, social-economic status, and position in the hierarchy. On the other hand, being a professor is still a rather different position than being a CEO who is responsible for a Fortune500 firm. To test whether a person is a member of a class of persons is essentially the same as the one underlying biometric facial comparison research aimed at the recognition of faces. We can therefore use the same likelihood ratio framework that underlies the modern biometric face comparison research.

The likelihood ratio that we will estimate is equal to the ratio of the probability of measuring appearance *x*, a particular facial pixel intensity measure, given that the class of the face is *c*, in casu CEO or a member of the benchmark group, and the probability of measuring that appearance for any class, that is across the total sample of faces of both CEOs and the benchmark. In order to do so, the means and co-variances of both class *c* and the total distribution will be estimated from training data. The appearance is represented by the intensities of the pixels in the face. These are collected in a vector *x* with dimension *N* (the number of pixels of a face). In our case, we normalised the images to 130x150 pixels, resulting in *N* = 19.500 pixels. Since this dimensionality of the facial appearances data is high relative to the number of faces in our sample, only the main elements of the covariance of the total distribution can be estimated reliably. For the chosen likelihood ratio based classifier, a common approach is to perform a principal component analysis (PCA). With the PCA the vector with facial appearances is transformed such that only the directions of the main variations remain which results in an effective dimensionality reduction. For the individual classes next the maximum discriminative directions are found using Linear Discriminant Analysis (LDA). The PCA-LDA classifier that we used performs two steps of dimension reduction. The first step, PCA, is just a linear transformation of the data that de-correlates it, and drops dimensions that contain no relevant information. In the second step, LDA, the dimensionality of the data are further reduced to a space with a dimensionality of at most the number of classes of the problem in which the separation between the classes is performed. In this case this dimensionality is two. Since this is far below the number of participants, there is no chance that the model is saturated. Note that we took care to separate the training and testing phases: performing PCA and LDA on the training data results in transformations to map the training samples to a lower dimensional training-PCA resp. training-LDA space. These transformations were then used on the test set to map the test samples into the same lower dimensional training-PCA resp. training-LDA space from which in the end the likelihood ratios are determined. For more statistical background of the likelihood ratio framework, we refer to S1 File [19].

We collected photos for all CEOs from the set of US Fortune500 firms (see www.fortune.com/fortune500) for the period 2008–2013. In order to have high quality material, we only selected pictures with sufficiently high resolution (at least 130x150 pixels) and photos with frontal faces only, so specific care was taken that all CEO images were comparable in quality to the images in the FRGC database of citizens (see below). For all selected images only minor facial expressions were present. We corrected for race and gender so that we restricted our data to white male CEOs only. This gave us 674 unique CEOs. We then cropped and normalized each photo [35]. Each picture was cropped by first locating the centers of the eyes, alignment by scaling, rotation and translation, such that the eyes are always in the same location and finally cropping using an elliptic mask, that removes all background and almost always also all hair, and only keeps the inner part of the face. Illumination was mostly homogeneous and variation in illumination was further suppressed by executing an intensity (grey level) normalisation, to make the appearance of the facial images better comparable, using histogram equalisation [42]. To emphasize the high contrast areas in the images, that contain most descriptive information, we scaled the gray levels with a factor of 3 and clipped at 255. Note that except for the localisation of the eyes, all steps in cropping and normalisation were performed automatically.

For the data set of US citizens we used facial images from the Face Recognition Grand Challenge (FRGC) dataset [43] and collected in total photos of 229 white males (in the same age range as the CEOs so that the average age of the citizens matched those of the CEOs). We also collected 252 photos for university professors from five US universities. Again, we excluded all female and non-white professors, and we also excluded professors from business schools in order to minimize the chance that we include professors who (also) have (had) a leadership position within firms. We found photos on the internet, and then again cropped and normalized each photo [35].

In total, we thus collected photos of 674 white male faces of CEOs, 229 white male citizens and 252 white male university professors. Moreover, we collected a number of associated firm specific variables from the set of US Fortune500 firms covering the period 2008–2013, that is profits (as % of total assets), profits (as % of total revenue), and total revenue. In our estimations, we control for industry effects by subtracting the industry average from a firm’s revenues and profits.

In order to investigate whether CEOs’ faces significantly differ from average citizens and professors, we (randomly) took 150 CEO photos out of our sample of 674 CEO photos and similarly (randomly) 150 photos of citizens, and 150 of professors. Since the amount of data is limited, these numbers were chosen to have available a reasonable number of images for training and evaluation. The training and evaluation sets should, of course, be disjunct. A larger training set may improve the performance of the classifier, but it would leave a smaller evaluation set and, hence, less accurate performance reporting. A larger evaluation set on the other hand, increases the accuracy of performance reporting, but fewer samples for training are left, which will result in a poorer classifier. The choice strikes a balance between the two. These three subsamples of CEOs, citizens and professors were used as our training data and via the PCA/LDA/likelihood ratio procedure outlined above we estimated the mean and the covariance of the distribution of class c (CEO or citizen or professor), and the mean and the covariance of the total distribution, so as to be able to calculate the likelihood ratio (for more background information, see S1 File). This estimation was then used in the second step to test the model by using the estimations to predict for the remaining 524 (= 674–150) CEOs and 79 (= 229–150) citizens and 102 (= 252–150) university professors, if the corresponding faces would fall into either the CEO or the citizen, in either the CEO or the university professor category, and again in either the citizen or the university professor category. We re-ran this procedure for the CEOs and the citizens three times, that is to say we performed the analysis for three different random compositions of the training groups of 150 CEOs and 150 citizens. To test whether any two groups (CEOs vs citizens, CEOs vs professors, citizens vs professors, ‘top’ CEOs vs ‘bottom’ CEOs etc.) differ significantly from each other, we applied the Mann-Whitney test.

## Results

**Fig 1:**
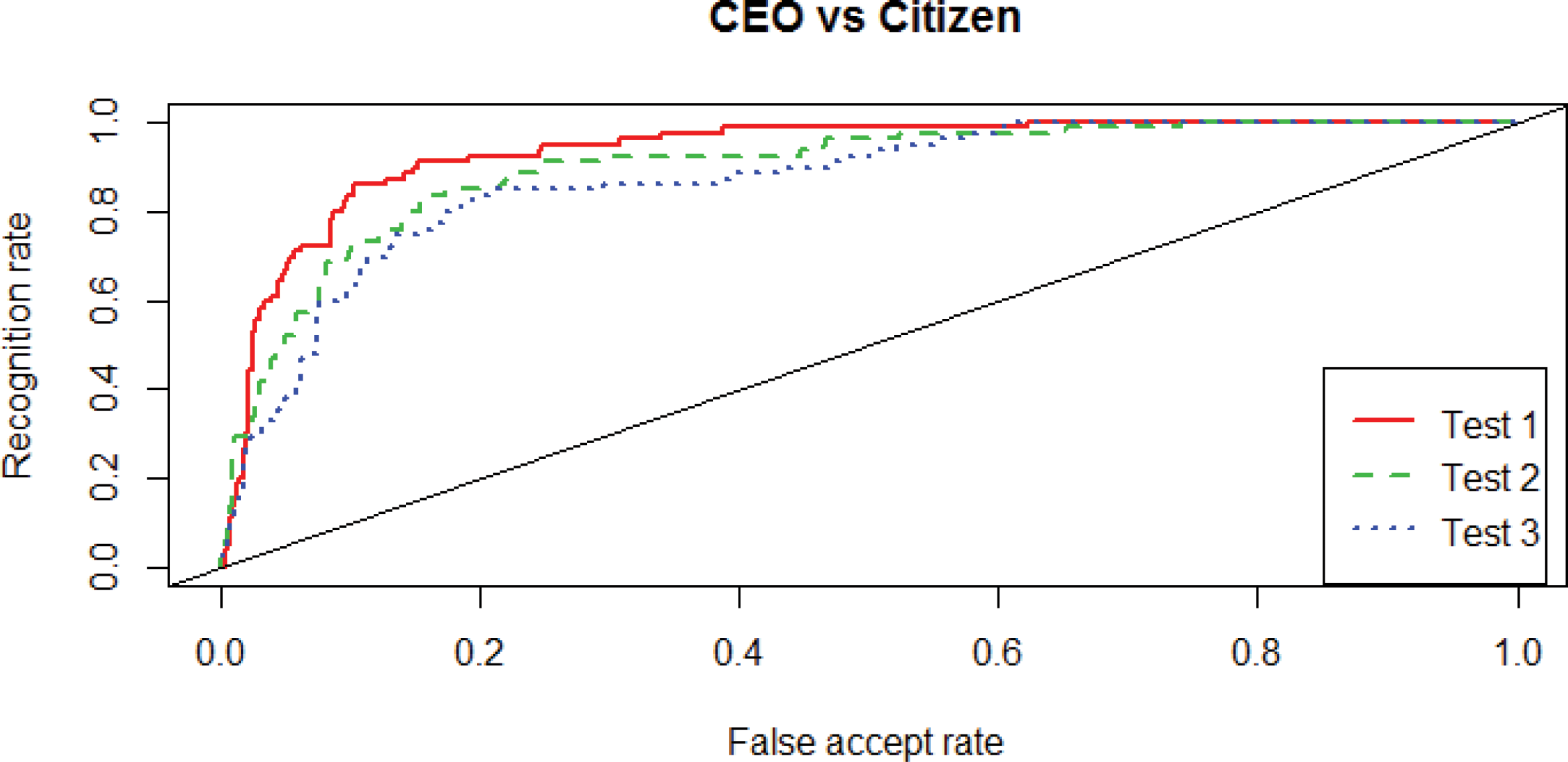
Faces of CEOs (in 3 test-groups, *n* = 524 in each test group) compared to citizens (*n* = 79 in each test group). Each test line represents a test for a different random composition of CEOs and citizens in the training and test group. For each test, the training group is 150 for CEOs and 150 for citizens, so the remaining faces fall into the test group of CEOs (*n* = 524) and citizens (*n* = 79).

To determine if a facial image represents a CEO or not, first the classifier determines the log of the likelihood ratio (LLR) by comparing the facial image to the average of the CEO faces. In order to decide if the facial image is a CEO or not, the LLR is compared to a threshold T. The class CEO is assigned if LLR>T. Fig 1 shows a so-called ROC (Receiver Operating Curve) for the three different compositions (Test 1, 2 and 3) of the classification of facial images as CEO. The ROC shows the TAR (True Accept Rate) on the vertical axis as a function of the FAR (False Accept Rate) on the horizontal axis for varying threshold T. The TAR is the rate at which CEO faces are correctly classified as CEO and equal to 1-FRR (False Reject Rate, type II error or β) which is the rate at which CEO faces are incorrectly classified as citizen. The FAR (False Accept Rate, type I error or α) is the rate at which citizen faces are incorrectly classified as CEO. If the threshold T is very high, no facial images are accepted as CEO and thus FAR = 0, but also TAR = 0. If on the other hand T is very low, all images are accepted as CEO, resulting in FAR = 1 and TAR = 1. In our set up, the TAR is the same as the positive predictive value (PPV), and (1-FAR) is the same as the negative predictive value (NPV). A perfect classifier would result in TAR = 1 at FAR = 0, i.e. the point in the upper left corner of the graph. If the actual test of the model would yield a range of false accept and accept rates that coincides with the diagonal in Fig 1, this would indicate that the estimated model would not have any predictive power. Hence, for the model to have predictive power in terms of Fig 1, the actual plot of the tested model should give true accept rates that significantly exceed the false accept rates.

Fig 1 suggests for our test group of 524 CEOs and 79 citizens that the faces of CEOs and citizens differ, since for any given false accept rate the corresponding recognition rate is clearly higher. This is confirmed by results for the Mann-Whitney test. In Table 1, the significant p-values reported for CEOs vs citizens for our three groups 1, 2, and 3 clearly indicate that the CEOs’ faces differ from those of the citizens’ class.

**Table 1:** Results of the Mann-Whitney test for the comparison of various classes. p = p-value.

*Selection*	*p-value*
CEO vs Citizens 1	p<2.2e-16***
CEO vs Citizens 2	p<2.2e-16***
CEO vs Citizens 3	p<2.2e-16***
CEO vs Professors	p = 9.09e-05***
Professors vs Citizens	p = 2.76e-10***

***p < .001

**Fig 2:**
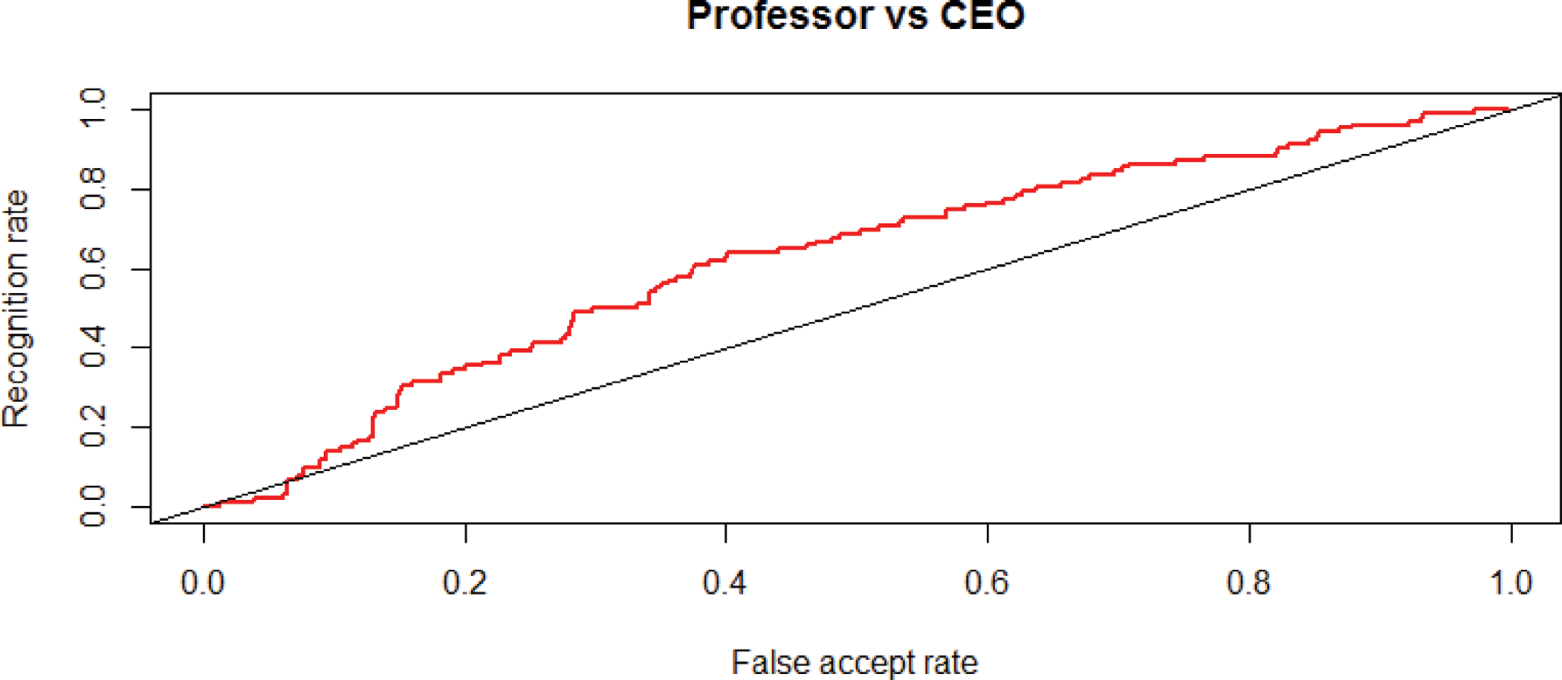
Faces of CEOs (*n* = 524) compared to Professors (*n* = 102) The red line represents a test for a random composition of CEOs and professors in the training and test group. For each test, the training group is 150 for CEOs and 150 for professors, so the remaining faces fall into the test group of CEOs (*n* = 524) and professors (*n* = 102).

In order to test whether the difference between CEOs and citizens might be explained by the fact that CEOs differ significantly in educational level, socio-economic status or position in the hierarchy, we also compared their faces with those of a comparable group of people, university professors. Fig 2 shows these results, and indicates that the faces of CEOs and professors also differ, but less strongly than the results for CEOs and citizens. This is confirmed by results for the Mann-Whitney test. In Table 1, the significant p-values reported for CEOs vs professors show that CEOs’ faces also differ from those of the professors group. Interestingly and in line with earlier findings on occupational stereotyping [25–26], the university professors also differ significantly from the citizens, as can be seen in the final row of Table 1.

One could also infer significance from the ROC-curves in Figs 1 and 2. Every point on the ROC curve in Figs 1 and 2 represent the FAR and TAR for a certain threshold on the comparison score. Of course, due to limited available testing data, there is an uncertainty in the FAR and TAR. If the same experiment were repeated with different data, slightly different FAR and TAR values would result. As a matter of fact, the FAR and TAR values follow binomial distributions B(N,m), where N is the number of CEO or citizen samples and m is the mean of the TAR or FAR. We calculated Jeffreys credible intervals, see [44, 45] which provide an estimate of the intervals in which the true FAR and TAR will lie. E.g. for FAR = 0.2 and TAR = 0.9 in Fig 1, the 95% Jeffreys credible intervals are (0.88–0.92) for the TAR and (0.16–0.25) for the FAR. This means that there is a 95% probability that the actual TAR is in the range (0.88–0.92) and the actual FAR in the range (0.16–0.25). This means that even in the worst case, if actual FAR is 0.25, the TAR at 0.88 is still far above gambling, which would mean a TAR = 0.25.

Consequently, and following previous studies that focus on the relevance of CEOs’ faces for firm performance [4, 6, 7], we investigated whether facial features of CEOs can predict actual performance. We created two groups of CEOs based on performance criteria (total revenue, profits (as % of total assets), and profits (as % of total revenue)), that is a group with the top-100 firms and their associated CEOs, and a group that consisted of the bottom-100 firms and their associated CEOs. The financial performance criteria are taken from the Fortune 500 website at http://fortune.com/fortune500/2012/. For this within-group CEO comparison we took the CEOs that were active as such in 2012, this gives us 481 CEOs from our sample of 674 unique CEOs. The reason to stick to 2012 is that we want to control for possible year or time effects. We controlled for firm size, so instead of taking total profits we took profits as a percentage of total assets or total revenue [7]. To estimate this ‘within group’ model, we first took for each of the three firm performance variables 80 CEO photos from each class and we then used the model estimations to test the model by using the estimated parameters to predict in which category (top or bottom) the remaining 20+20 CEOs would belong. The results of these analyses can be found in Table 2. Results show for all of our top vs bottom comparisons that the model is *not* able to differentiate between the faces of top100 or bottom100 CEOs, indicating that one can clearly not reject the hypothesis that the respective two groups of CEOs have similar faces.

**Table 2:** Results of the Mann-Whitney test for classes of Top vs Bottom CEOs. p = p-value.

*Performance criterium*	*p-value Top vs Bottom CEOs*
Profit/Assets	p = 0.3378
Profit/Revenues	p = 0.28
Revenues	p = 0.7354

## Discussion

Applied to a large sample of Fortune500 CEOs and by using an advanced objective method for the measurement of facial features, we find that US CEOs’ faces differ significantly from US citizens and university professors. Hereby we replicate the finding in facial appearance research at large [3, 8] that leaders also objectively somehow look different than non-leaders, thereby supporting the work on occupational stereotyping [25–26]. Applied to the case of CEOs, this is, however, the first study to objectively and comprehensively compare the group of CEOs with benchmark groups of citizens and professors, and thereby it supports the claim that selection of leaders is at least partly driven by their facial appearance.

More importantly, and here our research ties up with earlier, partly contradictory studies [4, 6, 7], our results suggest that CEOs’ faces do *not* relate to firm performance. The strength of our study is that it is based on an objective and more advanced method for measuring facial features than either the subjective lab-studies [4, 7] or the predetermined one-dimensional measures like fWHR or mouth width, which have been used so far in comparable studies [6, 14]. Additionally, we base our conclusions on a substantially larger sample of Fortune500 firms than related studies. Thirdly, like Graham et al [7] already argued, a number of studies on the relationship between CEO facial appearance and performance are problematic as they [4, 14] use unscaled measures of firm performance only [7], thereby confusing firm size with profitability.

In general, based on this objective measure, our results strongly support recent conclusions [3, 8] that there is no evidence for a relationship between faces and actual ability of leaders. Moreover in doing so, we did not only investigate a large group of CEOs, but we also introduced an objective ‘real world base-rate’ [46] by comparing faces of CEOs with those from citizens and professors. The theoretical implication of our paper is that predicting the selection of leaders based on their facial appearance is not necessarily informative about their subsequent effectiveness or performance, in contrast to what some evolutionary scholars suggest.

A notable limitation of our method is of course that we do not know precisely what facial attributes discern CEOs from non-CEOs, but the potential applications of the Likelihood Ratio method for these type of studies are very promising and we suggest that future research could look into this matter. Additionally, a second limitation is that, by way of counterfactual, one would like to know what firm performance would be if randomly chosen US citizens (or US university professors) would be put in charge of Fortune 500 firms, so as to learn if the significant differences in facial appearances between CEOs and citizens also transform into differences in performance. Although appealing as an experiment, this role switching is not very likely to occur in reality. A final possible limitation is that CEOs may somehow look different because of the fact that they have become a CEO. Rule and Ambady [9] also discuss this causality issue in a setting where respondents have to assess photos of CEOs. With these subjective verdicts, the causality issue seems, however, potentially more of a problem than with the kind of objective face recognition methods employed here.

For future research on the relevance of facial appearance for various social phenomena we believe that more use of biometric methods could yield new and robust insights. Clearly, using such an objective measure of facial characteristics circumvents the errors and shortcomings of human observations [3]. One of the plans for future research is to investigate other types of classes and also to investigate simultaneous separation of multiple classes. Second, information on the facial features that pre-dates the CEO appointment stemming from for instance college yearbook photos seems a promising avenue to explore the causality issue mentioned above [47–48].

Our study has also strong practical implications. Boards of directors and HR-professionals within and outside organizations should be aware of the fact that selection of CEOs is a process in which the looks of a (potential) candidate fundamentally matter. But even more importantly, they should realize that ‘what you see is not necessarily what you get’ and therefore do not put too much weight on these processes, since at least we find no evidence that the face of a CEO is a predictor of actual performance.

## Supporting Information

S1 File Background information on the likelihood ratio framework. (DOCX)

## References

[1] Poutvaara P (2014) Facial appearance and leadership: An overview and challenges for new research. Leadership Quarterly 25: 801–804.

[2] Re D, Rule NO (2015) Facial appearance and CEO performance. In: M. Fetscherin (Ed.), CEO Branding: Meaning, Measuring, Managing.

[3] Todorov A, Olivola CY, Dotsch R, Mende-Siedlecki P (2015) Social attributions from faces: Determinants, consequences, accuracy, and functional significance. Ann Rev Psychol 66: 519–545. 25196277 10.1146/annurev-psych-113011-143831

[4] Rule NO, Ambady N (2008) The face of Success. Inferences from Chief Executive Officers’ Appearance Predict Company Profits. Psychol Sci 19: 109–111. 10.1111/j.1467-9280.2008.02054.x 18271856

[5] Rule NO, Tskhay KO (2014) The influence of economic context on the relationship between chief executive officer facial appearance and company profits. Leadership Quarterly 25: 846–854.

[6] Wong EM, Ormiston ME, Haselhuhn MP (2011) ‘A face only an investor could love’ CEOs’ facial structure predicts their firms’ financial performance. Psychol Sci 22: 1478–1483. 10.1177/0956797611418838 22042727

[7] Graham JR, Harvey CR, Puri MA (2015) Corporate Beauty Contest. Available: http://ssrn.com/abstract=1571469 or 10.2139/ssrn.1571469.

[8] Olivola CY, Funk F, Todorov A (2014) Social attributions from faces bias human choices. Trends Cogn Sci 18: 566–570. 25344029 10.1016/j.tics.2014.09.007

[9] Rule NO, Ambady N (2009) She’s Got the Look: Inferences from Female Chief Executive Officers’ Faces Predict Their Success. Sex Roles 61: 644–652.

[10] Spisak BR (2012) The *General* Age of Leadership: Older-Looking Presidential Candidates Win Elections during War. PLoS ONE 7 (5): e36945 10.1371/journal.pone.0036945 22649504 PMC3359335

[11] Oosterhof NN, Todorov A (2008) The functional basis of face evaluation. Proc Natl Acad Sci 105: 11087–11092. 10.1073/pnas.0805664105 18685089 PMC2516255

[12] Antonakis J, Dalgas O (2009) Predicting elections: Child’s Play! Science 323: 1183 10.1126/science.1167748 19251621

[13] Said CP, Todorov A (2011) A statistical model of facial attractiveness. Psychol Sci 22: 1183–1190. 10.1177/0956797611419169 21852448

[14] Re DE, Rule NO (2015) The Big Man Has a Big Mouth: Mouth Width Correlates with Perceived Leadership Ability and Actual Leadership Performance. J Exp Soc Psychol (in press).

[15] Geniole SN, Denson TF, Dixson BJ, Carré JM, McCormick CM (2015) Evidence from meta-analyses of the facial width-to-height ratio as an evolved cue of threat. PloS ONE 10(7): e0132726 10.1371/journal.pone.0132726 26181579 PMC4504483

[16] Goldstein AJ, Harmon LD, Lesk AB (1971) Identification of human faces. Proc IEEE 59: 748–760.

[17] Turk M, Pentland A (1991) Eigenfaces for recognition. J Cogn Neurosci 3: 71–86. 10.1162/jocn.1991.3.1.71 23964806

[18] Zhao W, Chellappa R, Rosenfeld A, Phillips PJ (2003) Face recognition: A literature survey. ACM Computing Surveys 35: 399–458.

[19] Bazen AM, Veldhuis RNJ (2004) Likelihood ratio-based iometric verification. IEEE Transactions on Circuits and Systems for Video Technology 14: 86–94.

[20] Spreeuwers LJ (2011) Fast and accurate 3D face recognition. Using registration to an intrinsic coordinate system and fusion of multiple region classifiers. Int J Comp Vision 93: 389–414.

[21] Alrajih S, Ward J (2014) Increased facial width‐to‐height ratio and perceived dominance in the faces of the UK's leading business leaders. British J of Psychol 105(2): 153–161. 10.1111/bjop.12035 24754804

[22] Beggs JM, Doolittle DC (1993) Perceptions now and then of occupational sex typing: A replication of Shinar's 1975 study. J Appl Soc Psychol 23: 1435–1453.

[23] King EB, Mendoza SA, Madera JM, Hebl MR, Knight J L (2006) What's in a name? A multiracial investigation of the role of occupational stereotypes in selection decisions. J Appl Soc Psychol 36: 1145–1159.

[24] Johnson SK, Podratz KE, Dipboye RL, Gibbons E (2010) Physical attractiveness biases in ratings of employment suitability: Tracking down the “beauty is beastly” effect. J Soc Psychol 150: 301–318. 10.1080/00224540903365414 20575336

[25] Lipton JP, O’Connor M, Terry C, Bellamy E (1991) Neutral job titles and occupational stereotypes: When legal and psychological realities conflict. J Psychol 125: 129–151.

[26] Oldmeadow JA, Sutherland CAM, Young AW (2013) Facial stereotype visualization through image averaging. Soc Psychol Pers Sci 4: 615–623.

[27] Zebrowitz LA, Montepare JM (2015) Faces and First Impressions Handbook of Personality and Social Psychology, Vol. 1: Attitudes and Social Cognition. Ed. Bargh J. & Borgida G.. Washington, DC: American Psychological Association (forthcoming).

[28] Lord RG (1985) An information processing approach to social perceptions, leadership and behavioral measurement in organizations. Res Org Behav 7: 87–128.

[29] Cherulnik PD, Turns LC, Wilderman SK (1990) Physical Appearance and Leadership: Exploring The Role of Appearance‐Based Attribution in Leader Emergence. J Appl Soc Psychol 20: 1530–1539.

[30] Antonakis J, Jacquart P (2013) The far side of leadership: Rather difficult to face In: Bligh MC, Riggio RE, Exploring Distance in Leader-Follower Relationships: When Near is Far and Far is Near. Routledge, pp 155–187.

[31] Olivola CY, Todorov A (2010) Elected in 100 milliseconds: Appearance-based trait inference and voting. J Nonverb Beh 34: 83–110.

[32] Olivola CY, Eubanks DL, Lovelace JB (2014) The many (distinctive) faces of leadership: Inferring leadership domain from facial appearance. Leadership Quarterly 25: 817–834.

[33] Judge TA, Piccolo RF, Kosalka T (2009) The bright and dark side of leader traits: A review and theoretical extension of the leader trait paradigm. Leadership Quarterly 20: 855–875.

[34] Van Vugt M, Hogan R, Kaiser RB (2008) Leadership, followership, and evolution: Some lessons from the past. Am Psychol 63: 182–196. 10.1037/0003-066X.63.3.182 18377108

[35] Carré JM, McCormick CM, Mondloch CJ (2009) Facial structure is a reliable cue of aggressive behavior. Psychol Sci 20: 1194–1198. 10.1111/j.1467-9280.2009.02423.x 19686297

[36] Haselhuhn MP, Wong EM (2012) Bad to the bone: facial structure predicts unethical behaviour. Proc Roy Soc London B: Biol Sci 279 (1728): 571–576. 10.1098/rspb.2011.1193 PMC3234568 21733897

[37] Lefevre CE, Lewis GJ, Bates TC, Dzhelyova M, Coetzee V, Deary IJ, et al (2012) No evidence for sexual dimorphism of facial width-to-height ratio in four large adult samples. Evolution and Human Behavior, 33: 623–627.

[38] Galinsky AD, Jordan J, Sivanathan N (2008) Harnessing power to capture leadership. Leadership at the crossroads 1: 283–299.

[39] Todorov A, Mandisodza AN, Goren A, Hall CC (2005) Inferences of competence from faces predict election outcomes. Science 308: 1623–1626. 15947187 10.1126/science.1110589

[40] Hess U, Adams RB Jr., Kleck RE (2005) Who may frown and who should smile? Dominance, affiliation, and the display of happiness and anger. Cogn & Emot 19: 515–536.

[41] Antonakis J (2011) Predictors of leadership: The usual suspects and the suspect traits. Sage handbook of leadership, pp 269–285.

[42] Gonzales RC, Woods RE (2008) Digital image processing. Pearson Education, Inc., ISBN 0-13-505267-X.

[43] Phillips PJ, Flynn PJ, Scruggs T, Bowyer KW, Chang J, Hoffman K, et al (2005) Overview of the Face Recognition Grand Challenge. Proc 2005 IEEE Computer Society Conference on Computer Vision and Pattern Recognition (CVPR'05) 1: 947–954.

[44] Bishop CM (2006) Pattern recognition and machine learning (information science and statistics) Springer-Verlag, New York.

[45] Brown LD, Cai TT, Dasgupta A (2001) Interval estimation for a binomial proportion. Stat Sci 101–133.

[46] Olivola CY, Todorov A (2010) Fooled by first impressions? Reexamining the diagnostic value of appearance-based inferences. J Exp Soc Psychol 46: 315–324.

[47] Re DE, Tskhay KO, Tong MO, Wilson JP, Zhong CB, Rule NO (2015) Facing fate: Estimates of longevity from facial appearance and their underlying cues. Archives Sci Psychol 3: 30–36.

[48] Rule NO, Ambady N (2011) Judgments of power from college yearbook photos and later career success. Soc Psychol Pers Sci 2:154–158.

