# Supplementary material for: The Facial Appearance of CEOs: Faces Signal Selection but Not Performance
Source: PLoS One. 2016 Jul 27;11(7):e0159950. doi: 10.1371/journal.pone.0159950 (PMC4963019; doi:10.1371/journal.pone.0159950)
Supplement: S1 File — (DOCX) [file pone.0159950.s001.docx]

**Supporting Information: background on the likelihood ratio framework**

The appearance is represented by the intensities of the pixels in the face. These are collected in a vector $x$ with dimension N (the number of pixels of a face). In our case, we normalised the images to 130x150 pixels, resulting in N=19500**.**

The likelihood ratio for a class c of a vector $x$ is defined as:

$$LR\left( c | x \right)=\frac{p\left( x | c \right)}{p\left( x \right)}$$

If we assume both distributions are Gaussian:

$$p\left( x | c \right)=\frac{1}{\sqrt{\left( 2\pi\right)^{N}\left| C_{c} \right|}}e^{-\frac{1}{2}\left( x-\mu_{c} \right)^{T}C_{c}^{-1}\left( x-\mu_{c} \right)}$$

$$p\left( x \right)=\frac{1}{\sqrt{\left( 2\pi\right)^{N}\left| C_{t} \right|}}e^{-\frac{1}{2}\left( x-\mu_{t} \right)^{T}C_{t}^{-1}\left( x-\mu_{t} \right)}$$

with $\mu_{c}$ the mean and $C_{c}$ the covariance of the distribution of class c, and $\mu_{t}$ the mean and $C_{t}$ the covariance of the total distribution. Then the log of the likelihood ratio becomes:

$$LLR\left( c | x \right)=-\frac{1}{2}\log\left( \left| C_{c} \right| \right)+\frac{1}{2}\log\left( \left| C_{t} \right| \right)-\frac{1}{2}\left( x-\mu_{c} \right)^{T}C_{c}^{-1}\left( x-\mu_{c} \right)+\frac{1}{2}\left( x-\mu_{t} \right)^{T}C_{t}^{-1}\left( x-\mu_{t} \right)$$

Because the dimensionality of *x* is very high, all elements of the covariance matrices cannot be estimated reliably from limited data. We therefore first perform a dimension reduction using Principle Component Analysis on a set of mixed CEO facial images and citizens, where only the M largest eigenvalues and corresponding eigenvectors are retained. This results in a transformation *T* that transforms the N dimensional input vector to an M dimensional vector. In our research we used M=200.

In addition, the best separation between classes is obtained if only the components with the smallest variations within class c are retained (Linear Discriminant Analysis = LDA), resulting in a second dimensionality reduction.
